# Varenicline induced acute interstitial nephritis in the setting of idiopathic membranous glomerulonephritis

**DOI:** 10.1186/1471-2369-14-248

**Published:** 2013-11-11

**Authors:** Wilson Kwong, Christine A White

**Affiliations:** 1 Department of Undergraduate Medical Education, Queen’s University, 80 Barrie Street, Kingston, Ontario K7L 3 N6, Canada; 2 Division of Nephrology, Etherington Hall, Room #3048A, Queen’s University, 94 Stuart Street, Kingston, Ontario K7L 3 N6, Canada

**Keywords:** Acute interstitial nephritis, Varenicline, Smoking cessation, Drug induced renal toxicity

## Abstract

**Background:**

Varenicline is a nicotinic receptor partial agonist indicated for the cessation of smoking. It is regarded as having no or minimal renal toxicity. A single case report has linked it to acute interstitial nephritis.

**Case presentation:**

A 56 year-old female with a long-standing history of idiopathic membranous glomerulonephritis presented on routine follow-up with an unexpected rise in her serum creatinine from a stable baseline of 225 umol/L to 319 umol/L Biopsy revealed acute interstitial nephritis. There were no preceding clinical events other than the initiation of varenicline therapy three months prior. This was discontinued with no improvement in renal function. A ten week course of prednisone was initiated and creatinine levels returned to baseline. Shortly after prednisone therapy was completed, renal function worsened but the patient declined further immunosuppressive therapy. Exposure to varenicline therapy two years prior had also resulted in a reversible decline in kidney function.

**Conclusion:**

This is only the second case report to document varenicline-induced acute interstitial nephritis. A careful medication history and renal biopsy were essential in identifying the etiology of the acute kidney injury in this patient with a complex renal history.

## Background

Over the past 20 years, a variety of different options both pharmacologic and non-pharmacologic – have been proposed to facilitate the process of smoking cessation [1]. Varenicline (*Champix, Pfizer*) is a nicotinic receptor partial agonist with proven efficacy in facilitating smoking cessation [2]. Pharmacokinetic studies show a predominantly renal mechanism of drug clearance [3,4] and dosing must be adjusted accordingly to the glomerular filtration rate (GFR) [5].

While varenicline has recently been linked to an increase in cardiovascular side effects [5,6], it is not generally thought to have significant direct renal toxicity. The product monograph does not list any renal effects in its table of most common adverse reactions. Polyuria, glycosuria, nephrolithiasis, nocturia, urine abnormality, urethral syndrome, acute renal failure and urinary retention are included in the list of less common clinical trial adverse drug reactions [5]. Specifically, acute renal failure is cited as “rare” [5]. The major trials demonstrating the efficacy of varenicline in smoking cessation do not report any adverse renal events [7-9]. These trials evaluated a 1 mg twice-daily treatment regimen of varenicline (6-week treatment, n = 127; 12-week treatment, n = 344, 12-week treatment, n = 352), excluded patients with renal impairment (not specifically defined) and followed patients for 52 weeks after treatment cessation [7-9].

A detailed literature review has revealed only two case reports documenting potential renal injury [10;11]. The first reported a temporal association between varenicline use and undefined acute kidney injury [10], while the second reported the development of biopsy proven acute interstitial nephritis (AIN) in the setting of underlying hypertensive nephrosclerosis and exposure to varenicline [11].

In this current report, a patient with longstanding idiopathic membranous glomerulonephritis (MN) develops biopsy proven AIN shortly after starting varenicline. Kidney function did not improve with discontinuation of the varenicline but did subsequently after initiation of prednisone treatment. Exposure to varenicline 2 years prior had also resulted in a reversible decline in renal function. The patient provided consent for this case report.

## Case presentation

A 56 year-old female presented to Nephrology in 1993 with nephrotic syndrome (15 g/day proteinuria, peripheral edema and serum albumin of 14 g/L) and normal renal function (Cr 70 umol/L). Percutaneous renal biopsy was performed which was consistent with stage II MN. There were no tubular or interstitial infiltrates. Her MN was felt to be idiopathic. She was treated with prednisone for a total of 12 weeks with decline in proteinuria to 1.1 g/d. Over time, she experienced a gradual and partial relapse with fluctuating urinary protein excretion (2.5-7.5 g/day) managed using RAS blockade. This was associated with gradual elevation of her serum creatinine to 105 umol/L by April 2009. In June 2009, prednisone (6 month treatment period) was initiated along with cyclosporine. Although her proteinuria did improve, her creatinine slowly increased and the cyclosporine was eventually discontinued in March 2010 when her creatinine reached 200 umol/L. During therapy, trough cyclosporine levels were less than 130 ug/L.

**Figure 1:**
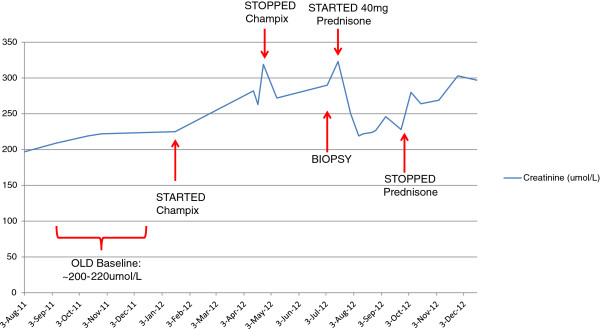
**Changes in serum Creatinine concentration (umol/L) Over Time; Creatinine levels increase substantially following treatment initiation with varenicline (200–220 umol/L to 319 umol/L).** Treatment cessation resulted in little improvement, but prednisone therapy lowered creatinine levels to ~228umol/L. Renal function continued to deteriorate after prednisone therapy was stopped.

In April 2012, at her routine nephrology clinic visit, she was found to have a Cr of 319 umol/L. Her creatinine over the past year had ranged between 200–220 umol/L. (Figure 1) She was clinically well and denied any recent illnesses, fevers, rashes, nausea, vomiting, diarrhea or reduced oral intake. She denied any flank pain, changes in her urination or shortness of breath. She had no symptoms of systemic vasculitis and had no constitutional symptoms. There were no recent changes in her prescription or over the counter medication, other than initiation of varenicline in Jan 2012 (Cr 225 umol/L). There was no non-steroidal anti-inflammatory (NSAID) medication or herbal remedy use.

Physical examination was unremarkable. She had no rashes, uveitis or dry eyes/mouth. Numerous granular casts were seen on urine microscopy. Other investigations including ANA, ENA antibodies (SSA/Ro, SSB/La, SM, RNP, Scl-70), RF, C3, C4, serum calcium, ANCA, HBV and HCV were all unremarkable. Peripheral blood eosinophil count was normal at 0.7 × 10^9^/L. Urinary protein to creatinine ratio was 0.76 g/mmol as compared to previous in May 2011 of 0.51 g/mmol. Ultrasound revealed normal sized kidneys (10.0 and 10.7 cm) with mildly increased echogenicity bilaterally. There was no evidence of renal vein thrombosis or hydronephrosis.

It was questioned whether the varenicline was the culprit and this was discontinued in late April 2012 (exact date unknown). As renal function did not improve and urine microscopy remained active, the patient eventually consented to renal biopsy. This was performed in late June 2012 and revealed 18 glomeruli, 3 of which were globally sclerotic. The glomeruli were normocellular but had marked thickening of the basement membrane with complex chain-like abnormalities on silver stain. There was moderate interstitial fibrosis and tubular atrophy. There were also patchy moderately intense interstitial inflammatory infiltrates of lymphocytes and eosinophils along with a lymphocytic tubulitis and acute tubular epithelial injury. There were no granulomas. Immunofluorescence showed diffuse moderate to extensive staining for IgG, C3, kappa and lambda. Electron microscopy was not performed.

When the results became available in early July 2012, prednisone therapy was initiated at 40 mg for 4 weeks. The dosage of prednisone was then tapered to 30 mg, followed by a decrease of 10 mg following each subsequent week of treatment. Once at 10 mg/day it was decreased by 2.5 mg on a weekly basis. In total, the full course of prednisone treatment lasted 10 weeks.

Renal function improved throughout the course of prednisone treatment, with creatinine levels returning to baseline (228 umol/L) by Sept 2012 and absence of any activity on urinalysis. Two weeks after prednisone cessation creatinine began climbing again gradually reaching 300 umol/L by November 2012. The patient declined repeat biopsy, re-initiation of prednisone or the introduction of alternate immunosuppressant agents. Her most recent creatinine value was 315 umol/L (March 2013).

Of interest, she had also taken varenicline for three months during the time of kidney function deterioration (January –March 2010) which had been attributed to cyclosporine. She stopped the varenicline and resumed smoking the same month the cyclosporine was discontinued (exact dates unknown to patient). A renal biopsy was not performed and there is no record of urine microscopy. By July 2010, creatinine levels had dropped to 140–160 umol/L from 200 umol/L (March 2010). Over the subsequent year kidney function slowly deteriorated with creatinine values remaining stable throughout 2011 at 200–220 umol/L.

Her past medical history otherwise includes hypertension, steroid-induced diabetes that has since resolved and remote knee surgery. She has never been diagnosed with an auto-immune/connective tissue disorder or sarcoidosis. She has never been hypercalcemic. Chest X-ray was unremarkable. She has no family history of renal disease. Her medications in April 2012 included darbepoetin 20mcg subcutaneously every 21 days, ferrous gluconate 900 mg per oral (po) daily, ASA 81 mg po daily, atorvastatin 20 mg po daily, and enalapril 15 mg po daily. She was not consuming any non-steroidal anti-inflammatory drugs (NSAIDS) and she had no recorded allergies. The enalapril was subsequently discontinued and replace with Cardizem CD.

## Conclusions

To our knowledge, this is only the second case report to document varenicline-induced AIN and the third describing any renal injury.

In 2009, Selby et al. [11] described a patient with CKD (baseline Cr of 124 umol/L) secondary to hypertension who presented with biopsy proven AIN (Cr 230 umol/L) seven months after initiating varenicline treatment. There were no other new medications. The patient was symptomatic with malaise and low grade fevers. A short course of prednisone therapy (60 mg for 10 days) was initiated with improvement in serum creatinine levels to slightly above baseline levels (Cr 160 umol/L) within three weeks of treatment initiation. Follow-up creatinine levels were not provided. Bird et al. [10] also reported varenicline-induced acute renal failure in a patient with moderate renal impairment. A diagnosis of AIN was postulated and renal function improved upon cessation of the medication. Renal biopsy was not performed.

AIN represents approximately 15-27% of acute kidney failure presentations, but may be under-represented since not all patients suspected of AIN are sent for renal biopsies [12]. AIN is characterized by inflammation of the kidney interstitium and a corresponding decline in renal function [13]. On biopsy, interstitial infiltrates consisting of lymphocytes, macrophages and eosinophils and tubulitis are seen [13]. AIN can be caused by medications, infections (viral, bacterial and fungal), systemic diseases (sarcoidosis, lupus erythematosus) or be idiopathic in nature [13]. Of all these causes, drug-induced AIN is the most common, accounting for up to 75% of all documented cases [14,15]. Drugs most known to induce AIN include certain classes of antibiotics and NSAIDs although a wide variety of other medications have also been implicated [15].

The clinical presentation is highly varied with the classic triad of fever, rash and eosinophilia occurring in a small minority of patients [16]. Non-beta lactam drug induced AIN is associated with eosinophilia in fewer than 1/3 of cases and eosinophiliuria in 2/3 of cases [15]. Thus renal biopsy is required for a definitive diagnosis [15]. Management consists of discontinuation of the culprit medication. The role of steroids is not clear and there are no randomized controlled studies to guide therapy [15]. In some [17,18], but not all [16,19] uncontrolled reports, steroids appear to have some beneficial role [18,19]. There is a suggestion that prognosis is much improved with early identification of the AIN and institution of steroid therapy [18].

Our patient did not have any clinical, serologic or radiographic evidence of autoimmune/systemic diseases such as SLE, Sjogren’s syndrome and sarcoidosis. There was no evidence to suggest infection. With the exception of varenicline which was prescribed approximately 4 months prior to the diagnosis of AIN the patient had been taking all other medications for a prolonged period of time. Thus the Varenicline seems the most likely culprit for her AIN.

The lack of improvement in renal function with cessation of varenicline is not inconsistent with drug-induced AIN. In one case series, 37% of patients with drug-induced AIN did not recover with medication withdrawal [17]. All of these patients experienced improvement in kidney function with steroid initiation [17]. Our patient’s renal function did improve back to her baseline with steroid therapy demonstrating reversibility of the active lesion despite the underlying chronic disease. Worsening of renal function after steroid withdrawal has also been described in the literature [20]. Preddie et al. describe two patients with acute kidney injury and AIN on renal biopsy attributed to medications (ciprofloxacin and unspecified medication). Both were steroid dependent and experienced renal recovery with mycophenolate mofetil (MMF) therapy [20]. Our patient declined further therapy with steroids or MMF.

Interestingly, her 3 month exposure to varenicline in 2010 was also accompanied by a reversible decline in GFR although there is no pathologic confirmation of AIN at that time and other factors such as discontinuation of the cyclosporine could also reasonably be implicated.

The other case report of AIN attributed to varenicline [11] also occurred in a patient with pre-existing renal disease. Drug-induced AIN is mediated by immune reactions initiated by drug deposited in the interstitium or drug processed and expressed by tubular cells acting as antigens and triggering both cell-mediated (predominantly) and humoral responses [16]. It has been hypothesized that counter-regulatory mechanisms involving suppressor T cells counterbalance the nephritogenic antigens [13]. When these protective mechanisms fail, AIN develops. Genetic susceptibility with deficient protective mechanisms has been proposed as one reason why AIN only develops in a minority of patients [13]. It also conceivable that, in the presence of ongoing renal disease and inflammation, there may be disruption in the normal counter-regulatory mechanisms thereby predisposing such patients to AIN. Further study is required.

In conclusion, we describe a case of biopsy proven AIN in the setting of varenicline use which did not improve with drug cessation but did with corticosteroid therapy. The patient relapsed after steroid withdrawal and declined further immunotherapy. This is only the second case of AIN described secondary to this smoking cessation aid. Similarly to the first case report [11] the subject had underlying renal disease. A thorough assessment including medication history and renal biopsy was helpful in establishing the diagnosis in this patient with a complex past renal history.

## Consent

Written informed consent was obtained from the patient for publication of this Case Report and any accompanying images. A copy of the written consent is available for review by the Editor of this journal.

## Abbreviations

AIN: Acute interstitial nephritis; MN: Membranous glomerulonephritis; GFR: Glomerular filtration rate; NSAID: Non-steroidal anti-inflammatory; MMF: Mycophenolate mofetil.

## Competing interests

The authors declare that they have no competing interests.

## Authors’ contributions

WK and CW were both involved with abstracting patient information from various hospital and clinic charts. CW obtained additional information directly from the patient through clinic visits and telephone interviews. WK and CW both helped draft, read and approve the final manuscript.

## Pre-publication history

The pre-publication history for this paper can be accessed here:

http://www.biomedcentral.com/1471-2369/14/248/prepub
